# SLC12A7 serves as a prognostic and immunotherapeutic biomarker identified by multi-omics analysis

**DOI:** 10.3389/fimmu.2025.1712579

**Published:** 2026-01-26

**Authors:** Haoran Qu, Jun Sun, Guohao Wang, Shuhong Ding, Xiaoming Li

**Affiliations:** 1 Department of Hepatobiliary and Pancreatic Surgery, The Second Qilu Hospital of Shandong University, Jinan, China; 2 Shandong Province Engineering Research Center for Multidisciplinary Research on Hepatobiliary and Pancreatic Malignant Tumors, Jinan, China

**Keywords:** biomarker, hepatocellular carcinoma, pan-cancer, scRNA-seq, SLC12A7, tumor immune microenvironment

## Abstract

**Background:**

Solute carrier family 12 member 7 (SLC12A7) is a membrane transporter implicated in ion homeostasis. Recent studies have suggested its aberrant expression in various malignancies; however, its functional roles and clinical relevance across cancers remain poorly defined. Given the limited understanding of SLC12A7 in oncogenesis and the urgent need for novel biomarkers, particularly in hepatocellular carcinoma (HCC), we conducted a comprehensive pan-cancer analysis to elucidate its potential oncogenic roles.

**Methods:**

Transcriptomic and clinical data from The Cancer Genome Atlas (TCGA), Genotype-Tissue Expression (GTEx), cBioPortal, TIMER 2.0, and Gene Expression Omnibus (GEO) were integrated to investigate the expression patterns, prognostic significance, genetic alterations, and immune correlations of SLC12A7. Single-cell RNA sequencing data were analyzed to examine its heterogeneity within the tumor microenvironment. Functional enrichment analyses were performed to identify associated signaling pathways. *In vitro* experiments were conducted to validate the effects of SLC12A7 on proliferation, migration, and invasion in HCC cell lines.

**Results:**

SLC12A7 was significantly upregulated in multiple cancer types and correlated with unfavorable clinical outcomes. Single-cell analysis revealed its enrichment in malignant cell populations and its involvement in the regulation of the tumor microenvironment. SLC12A7 expression was associated with immune cell infiltration and immune checkpoint gene expression. Functional assays confirmed that SLC12A7 enhances HCC cell proliferation, migration, and invasion.

**Conclusions:**

These findings identify SLC12A7 as a potential prognostic biomarker and immunotherapeutic target across cancers. Its oncogenic role in HCC suggests clinical utility in guiding prognosis and treatment strategies.

## 1 Introduction

Cancer is a serious global public health issue and has become the second leading cause of death worldwide, after cardiovascular diseases (1, 2). The International Agency for Research on Cancer (IARC) estimates that by 2040, the global cancer burden will reach 28.4 million cases. In China, HCC ranked third in newly diagnosed cancer cases and second in cancer-related deaths in 2022 (3). Different combination strategies of surgery, radiation therapy, chemotherapy, and other treatment options have been developed, significantly improving cancer treatment outcomes and extending survival. However, patients still face the risk of recurrence and metastasis. For instance, the five-year survival rate for HCC patients remains as low as 5%-30% (4). Therefore, sustained efforts are still required to discover new cancer biomarkers. Pan-cancer studies have become a vital approach for identifying common biomarkers that drive the occurrence and progression of diverse malignancies.

SLC12A7 is a member of the SLC12 gene family and is a transmembrane protein consisting of 1,083 amino acids. Its main biological function is to regulate cell volume through the transport of potassium and chloride. However, recent studies have reported that SLC12A7 is involved in the occurrence and progression of several types of cancer. SLC12A7 mediates tumor cell invasion by interacting with the membrane cytoskeleton/extracellular matrix junction protein Ezrin (EZR) (5). In cervical cancer, ovarian cancer, and breast cancer, overexpression of SLC12A7 is associated with local tumor infiltration, lymph node metastasis, and poor clinical outcomes (6). This suggests that SLC12A7 may be a potential therapeutic target for cancer. However, research on SLC12A7 in cancer is still very limited, with only a few studies reporting its function. To fill this gap, further exploration of its role and mechanisms in pan-cancer is necessary.

This study systematically analyzed the role of SLC12A7 across multiple cancer types using integrative multi-omics and single-cell analysis based on various databases. We assessed the expression levels of SLC12A7 in various cancers and its prognostic significance. scRNA-seq analysis revealed the heterogeneity of SLC12A7 in the tumor microenvironment and explored its role in tumor cells and macrophages. We explored the correlation between SLC12A7 and immune cell infiltration, immune checkpoint genes (ICKs), and genetic alterations. Additionally, we performed functional enrichment analysis with SLC12A7 to uncover potential molecular mechanisms that contribute to the development of multiple cancers. Extensive *in vitro* experiments validated the biological functions and mechanisms of SLC12A7 in liver cancer initiation and progression. These studies establish SLC12A7 as an independent prognostic biomarker and provide valuable insights for future research.

## 2 Materials and methods

### 2.1 Data source

Two HCC-related datasets [GSE14520 (7) and GSE76427 (8)] were obtained from the GEO database to validate the expression of SLC12A7 in HCC and normal liver tissues. Pan-cancer mRNA expression data, DNA methylation profiles, phenotypic data, survival data from TCGA, and normal tissue data from the GTEx database were downloaded via the UCSC Xena browser (https://xenabrowser.net/). Additionally, proteomic data across multiple cancer types were retrieved from the Clinical Proteomic Tumor Analysis Consortium (CPTAC) database (https://proteomics.cancer.gov/programs/cptac). Expression data for various cancer cell lines were obtained from the Cancer Cell Line Encyclopedia (CCLE, https://sites.broadinstitute.org/ccle/). Pan-cancer immune cell infiltration data were acquired from the Tumor Immune Estimation Resource 2.0 (TIMER2.0, http://timer.cistrome.org/). We also utilized the “Gene_DE” module of the TIMER2 platform to analyze the expression levels of SLC12A7 in various tumor types and their adjacent normal tissues from the TCGA project. The Tumor Immune Single-Cell Hub (TISCH, https://tisch.compbio.cn/home/) is a comprehensive database that integrates single-cell transcriptomic profiles from approximately 2 million cells across 76 high-quality tumor datasets, covering 27 cancer types (9). We utilized TISCH to investigate SLC12A7 expression across various tumors. Expression data for SLC12A7 at the single-cell level were retrieved from 12 datasets representing different tumor types and were visualized using UMAP plots for intuitive interpretation. Using GEPIA2 (GEPIA2, http://gepia2.cancer-pku.cn/), we analyzed the expression of SLC12A7 across different pathological stages (I-IV) in certain cancer types (10).

### 2.2 Single-cell transcriptome analysis

All analyses in the present study were performed using R software (version 4.1.1). The Single-cell RNA sequencing (scRNA-seq) data were analyzed using Seurat package (version 4.2) (11). Cells with fewer than 500 genes and fewer than 1,000 total RNA counts were excluded from further analysis. We used harmony to correct for batch effects across datasets. Principal component analysis (PCA) was performed with the highly variable genes after Z-score normalization. Uniform manifold approximation and projection (UMAP) dimension reduction was performed with the top 20 significant principal components (PCs). Clusters were determined using the FindClusters function (resolution = 0.6).

### 2.3 Prognosis analysis

The survival information of pan-cancer, including overall survival (OS), disease-specific survival (DSS), disease-free interval (DFI) and progression-free interval (PFI), was downloaded from TCGA database for evaluating the prognostic significance of SLC12A7. The prognostic significance of SLC12A7 in various cancers was evaluated using the Cox analysis and Kaplan-Meier model. Based on the median expression level of SLC12A7, all patients of each cancer type were divided into SLC12A7-high and SLC12A7-low groups. The R packages ‘survival’ and ‘survminer’ were used to perform Cox analysis and generate Kaplan-Meier (KM) survival curves to analyze the association between SLC12A7 expression and patient prognosis.

### 2.4 Immune cell infiltration analysis and immune checkpoint gene correlation analysis

The immune cell infiltration data for each cancer type were downloaded from the TIMER2.0 database (https://timer.cistrome.org/) (12). Spearman correlation analysis was used to assess the relationship between SLC12A7 expression and various immune cell subsets, and the results were visualized using heatmaps. A total of 11 immune checkpoint genes (including *PDCD1, CTLA4, VSIR, HAVCR2, LAG3, TIGIT, SIRPA, BTLA, SIGLEC7, LILRB2*, and *LILRB4*) were extracted from the TCGA dataset (13). Spearman correlation analysis was performed to evaluate the correlation between SLC12A7 expression and multiple immune checkpoint genes, and the results were visualized using heatmaps.

### 2.5 Microsatellite instability and tumor mutation burden analysis

TMB and MSI-related data were obtained from the cBioPortal database. Pearson correlation analysis was used to assess the correlation between SLC12A7 expression and TMB and MSI across multiple cancer types. The results were visualized using the online analysis platform (https://www.bioinformatics.com.cn), which enables the analysis and visualization of multi-omics data (14).

### 2.6 Gene set enrichment analysis

To assess the biological function of SLC12A7 in tumors, Pearson correlation analysis was performed to examine the relationship between SLC12A7 expression and other mRNAs retrieved from the TCGA transcriptome data. Based on the correlation index between genes and SLC12A7, genes most strongly correlated with SLC12A7 expression were selected for enrichment analysis. GSEA was performed using predefined gene sets from the Molecular Signatures Database and was conducted through the R package “clusterProfiler” (15). This study utilized the ‘c2.cp.kegg.v7.5.1.entrez.gmt’ and ‘c5.go.bp.v7.5.1.entrez.gmt’ gene sets for the GSEA analysis.

### 2.7 Genetic alteration analysis

Pearson correlation analysis was used to explore the relationship between SLC12A7 expression and methylation probe values, and the results were visualized using a heatmap. The genomic alterations of SLC12A7 were analyzed using the online database cBioPortal for Cancer Genomics (http://cbioportal.org), and the results were visualized with bar charts. Additionally, we used the online tool from cBioPortal to plot the prognostic differences between the SLC12A7 altered and unaltered groups. The copy number variation of SLC12A7 expression was assessed using the GISTIC 2.0 method.

### 2.8 Cell culture

The human HCC cell lines were procured from the Cell Bank of the Chinese Academy of Sciences (Shanghai, China). Cells were maintained in Dulbecco’s Modified Eagle Medium (DMEM; Gibco, Thermo Fisher Scientific) supplemented with 10% fetal bovine serum (FBS; Procell, China) and 1% penicillin-streptomycin solution under standardized conditions (37°C, 5% CO_2_ humidified atmosphere). Hypoxic experiments were conducted using a specialized hypoxia chamber, while other parameters remained consistent with normoxic culture. All cell lines underwent rigorous authentication via short tandem repeat (STR) profiling and mycoplasma contamination screening to confirm genetic integrity and sterility.

### 2.9 Plasmid construction and stable cell line generation

Expression plasmids (SLC12A7 overexpression, and knockout constructs) were sourced from General Biosystems (Anhui, China). Transient transfection of HCC cells was performed using Lipofectamine 3000 and P3000 enhancer (Thermo Fisher Scientific, USA). For lentiviral transduction, Huh7 cells (cultured in 10 cm dishes at 90% confluency) were co-transfected with SLC12A7 expression/knockout plasmids and packaging vectors (PMD2G/PSPAX2; Biosharp, China) using polyethyleneimine (PEI). Viral supernatants were harvested 48 h post-transfection, filtered (0.45 μm), and used to infect target cells. Stable clones were selected under 4 μg/mL puromycin pressure for 72 h, followed by validation via quantitative polymerase chain reaction (qPCR) and immunoblotting.

### 2.10 RNA isolation and qPCR analysis

Total RNA was isolated from HCC cells or tissues using TRIzol reagent (Vazyme, China) following chloroform phase separation and isopropanol precipitation. RNA pellets were washed with 75% ethanol, air-dried, and resuspended in DEPC-treated water. Purity and concentration were quantified spectrophotometrically (NanoDrop) with A260/A280 ratios maintained at 1.8–2.0. Reverse transcription was performed with 1 μg RNA using HiScript II Q Select RT SuperMix (Vazyme). qPCR amplification utilized Taq Pro Universal SYBR Master Mix (Vazyme) on a Thermo Fisher QuantStudio system under standardized cycling parameters: 95°C for 30 s, followed by 40 cycles of 95°C (15 s) and 60°C (30 s). Relative gene expression was normalized to GAPDH and calculated via the 2^−ΔΔCt method with triplicate technical replicates.

### 2.11 Western blotting assay

Protein lysates were extracted using RIPA buffer containing protease/phosphatase inhibitors (Beyotime, China), quantified via BCA assay, and denatured (95°C, 5 min). Equal protein loads (20–40 μg) were resolved on SDS-PAGE gels (80V for 30 min, 120V for 60 min) and transferred to PVDF membranes (300 mA, 105 min). After blocking with 5% non-fat milk, membranes were incubated overnight at 4°C with primary antibodies (SLC12A7 and β-actin), followed by HRP-conjugated secondary antibodies (1 h, RT). Protein bands were visualized using ECL substrate (Tanon, China) and quantified via ImageJ.

### 2.12 Cell proliferation and clonogenicity assays

For CCK-8 analysis, cells (2×10³/well in 96-well plates) were incubated with 10 μL CCK-8 reagent (Vazyme) for 1–2 h at 37°C. Absorbance (450 nm) was measured using a microplate reader at 24, 48, and 72 h. Colony formation assays involved seeding 500 cells/well in 6-well plates for 10–14 days. Colonies were fixed (4% paraformaldehyde), stained (0.1% crystal violet), and manually counted.

### 2.13 EdU incorporation and transwell migration/invasion

EdU-positive cells were detected using a BeyoClick™ EdU Kit (Beyotime). Briefly, cells were labeled with 10 μM EdU for 2 h, fixed, permeabilized, and stained with Alexa Fluor 594. Nuclei were counterstained with DAPI. Fluorescence images were acquired using an Olympus IX73 microscope. For Transwell assays, 5×10^4^ cells in serum-free medium were seeded into Matrigel-coated (invasion) or uncoated (migration) chambers (8 μm pores; Corning). After 36 h, transmigrated cells were fixed, stained, and quantified microscopically.

### 2.14 Scratch wound healing assay

Confluent monolayers in 6-well plates were scratched using sterile pipette tips. Debris was removed by PBS washing, and cells were cultured in serum-free medium. Wound closure was monitored at 0, 24, and 48 h using phase-contrast microscopy (Nikon Eclipse Ti). Migration rates were calculated via ImageJ-based analysis of wound area reduction.

### 2.15 Statistical analysis

Spearman correlation analysis was performed to explore: (1) The correlation between the expression of SLC12A7 and immune cell infiltration scores in pan-cancer patients from the TCGA database. (2) The correlation between the expression of SLC12A7 and immune checkpoint genes in pan-cancer patients from the TCGA database. Pearson correlation analysis was employed to investigate: (1) The correlation between SLC12A7 expression and methylation levels. (2) The pan-cancer expression of SLC12A7 in both TCGA and CCLE databases, as well as its expression in normal tissues from the GTEx database. Student’s t-test or one-way ANOVA was used to analyze inter-group differences as appropriate. All statistical analyses of *in vitro* experimental data were conducted using GraphPad Prism 9.0, with data presented as the mean ± standard deviation from ≥3 independent experiments. Statistical significance was defined as * P < 0.05, ** P < 0.01, and *** P < 0.001.

## 3 Results

### 3.1 SLC12A7 is significantly upregulated in multiple cancer types

**Figure 1:**
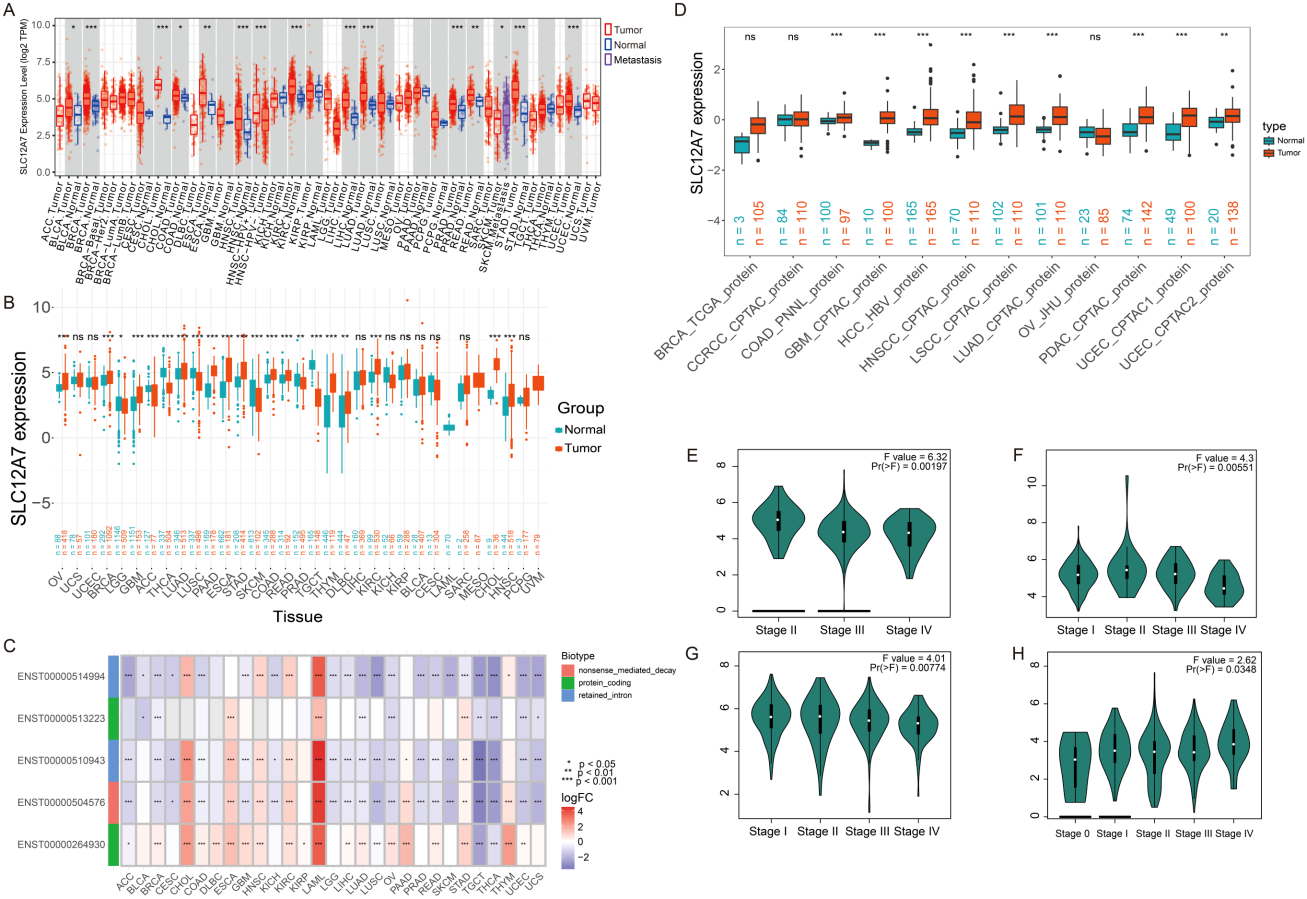
SLC12A7 is upregulated in multiple cancer types. **(A)** SLC12A7 mRNA expression in multiple cancers based on TCGA database. **(B)***SLC12A7* mRNA expression in 33 cancer types based on TCGA and GTEx database. The red and blue dots refer to tumor and normal tissues, respectively. **(C)** The non-coding transcripts demonstrated consistent downregulation in various cancer types. Red and blue squares represent upregulation and downregulation, respectively. **(D)** SLC12A7 protein expression in 11 cancer types based on the CPTAC database. E-H the correlation between SLC12A7 expression and the pathological stages of KIRC, KIRP, OV, SKCM. *p < 0.05; **p < 0.01; ***p < 0.001.

To investigate the expression pattern of SLC12A7 across pan-cancer, we analyzed transcriptomic data from TCGA and found that SLC12A7 was significantly upregulated in 13 cancer types, including bladder cancer (BLCA), breast cancer (BRCA), cholangiocarcinoma (CHOL), colon cancer (COAD), esophageal cancer (ESCA), head and neck cancer (HNSC), kidney cancer (KIRC), liver cancer (LIHC), lung adenocarcinoma (LUAD), prostate cancer (PRAD), rectal cancer (READ), gastric cancer (STAD), and uterine cancer (UCEC) (**Figure 1A**). Further integration of TCGA and GTEx datasets confirmed the significant upregulation of SLC12A7 in 15 cancer types, including ovarian serous cystadenocarcinoma (OV), BRCA, glioblastoma multiforme (GBM), adrenocortical carcinoma (ACC), LUAD, pancreatic adenocarcinoma (PAAD), ESCA, STAD, COAD, READ, thymoma (THYM), diffuse large B-cell lymphoma (DLBC), KIRC, CHOL, and HNSC (**Figure 1B**). In contrast, SLC12A7 expression was markedly downregulated in eight cancer types, including lower grade glioma (LGG), thyroid carcinoma (THCA), lung squamous cell carcinoma (LUSC), PRAD, and testicular germ cell tumors (TGCT) (**Figure 1B**). To further characterize the expression profile of SLC12A7, we analyzed five known transcript variants, including two protein-coding transcripts, two non-coding transcripts, and one transcript subjected to nonsense-mediated decay (NMD). Notably, one protein-coding transcript was significantly upregulated in multiple cancer types, whereas non-coding transcripts exhibited consistent downregulation across various malignancies (**Figure 1C**). At the protein level, analysis of CPTAC datasets from nine datasets representing 11 cancer types revealed that SLC12A7 protein expression was elevated in eight cancer types, including COAD, GBM, hepatocellular carcinoma (HCC), head and neck squamous cell carcinoma (HNSC), LUSC, LUAD, PAAD, and UCEC (**Figure 1D**). Moreover, GEPIA2-based analysis showed that SLC12A7 expression was significantly associated with tumor stage in four cancer types: KIRC (**Figure 1E**), KIRP (**Figure 1F**), OV (**Figure 1G**), and skin cutaneous melanoma (SKCM) (**Figure 1H**), suggesting a potential role in cancer progression and staging. Taken together, SLC12A7 exhibits consistently elevated expression across multiple datasets, including TCGA, GTEx, and CPTAC, at both transcriptomic and proteomic levels. This concordance strengthens the reliability of our findings and highlights the aberrant expression of SLC12A7 in various cancers, suggesting its potential involvement in tumor initiation and progression.

### 3.2 SLC12A7 is a prognostic pan-cancer biomarker

**Figure 2:**
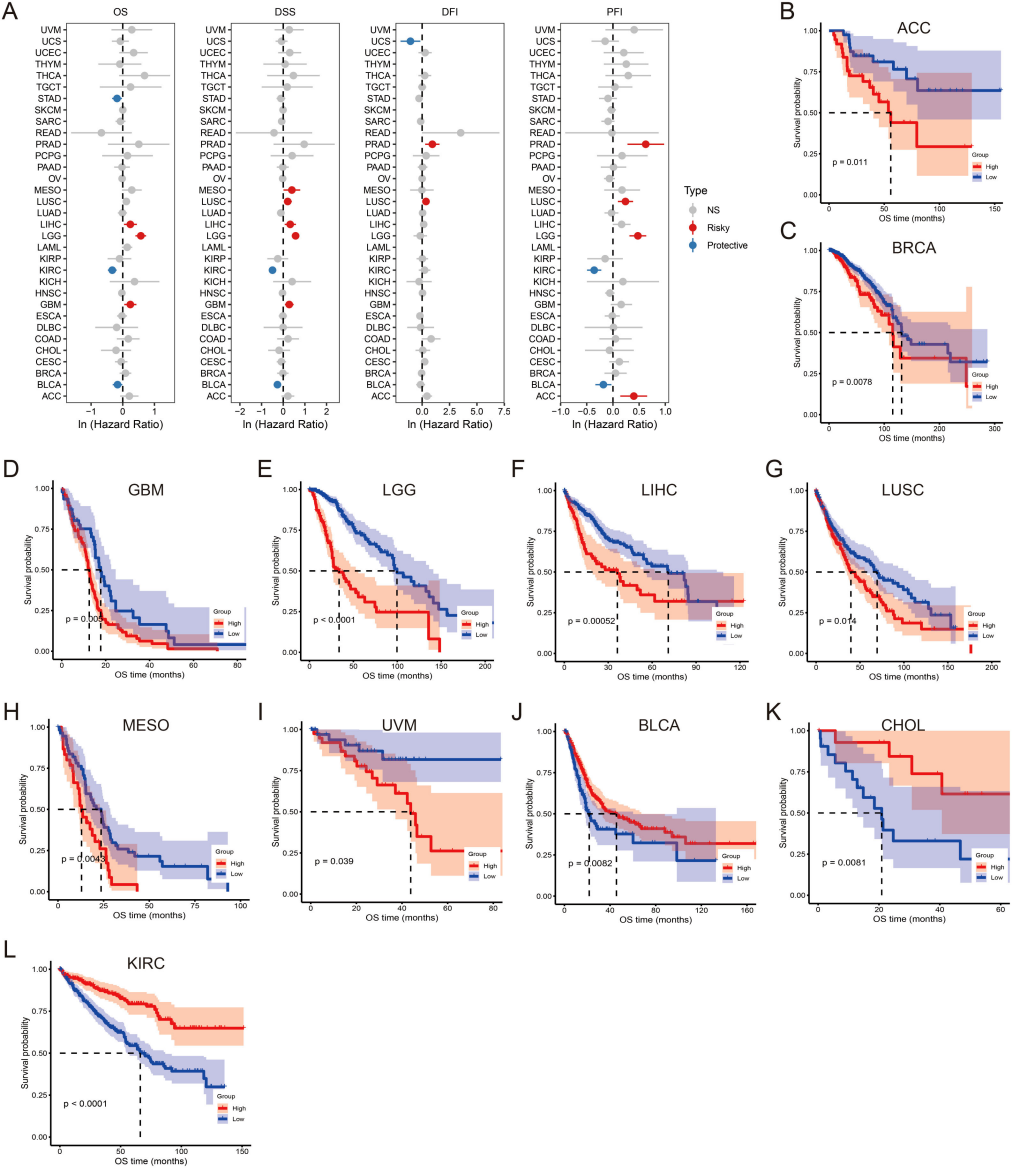
SLC12A7 is associated with poor OS, DSS, DFI and PFI. **(A)** The forest plot shows the univariable Cox regression analysis of OS, DSS, DFI and PFI based on SLC12A7 expression in 33 TCGA cancer types. **(B-L)** Kaplan-Meier analysis of OS for SLC12A7 mRNA expression in patients with ACC **(B)**, BRCA **(C)**, GBM **(D)**, LGG **(E)**, LIHC **(F)**, LUSC **(G)**, MESO **(H)**, UVM **(I)**, BLCA **(J)**, CHOL **(K)**,and KIRC **(L)** is also presented. NS, not significant.

To explore the clinical significance of SLC12A7 expression, Cox regression analysis was conducted in the TCGA database to examine the association between SLC12A7 expression and patient overall survival (OS), disease-specific survival (DSS), disease-free interval (DFI) and progression-free interval (PFI) across 33 cancer types (**Figure 2A**). The study found that high SLC12A7 expression was a risk factor for OS in LIHC, LGG, and GBM, while it acted as a protective factor in STAD, KIRC, and BLCA. In DSS, high SLC12A7 expression was a risk factor for MESO, LUSC, LIHC, LGG, and GBM, while it served as a protective factor in KIRC and BLCA (**Figure 2A**). Meanwhile, elevated expression of SLC12A7 was identified as a risk factor for DFI in patients with PRAD and MESO, whereas it served as a protective factor in UCS (**Figure 2A**). Regarding PFI, high SLC12A7 expression was associated with worse prognosis in ACC and LGG, while indicating a favorable outcome in KIRC and BLCA (**Figure 2A**). Notably, regardless of OS or DSS, high SLC12A7 expression was consistently a risk factor in LIHC, LGG, and GBM, whereas it was a protective factor in KIRC and BLCA. Kaplan-Meier survival analysis further revealed that, at the OS level, high SLC12A7 expression was significantly associated with poor prognosis in patients with ACC, BRCA, GBM, LGG, LIHC, LUSC, MESO, and UVM (**Figures 2B–I**). In contrast, high SLC12A7 expression was associated with favorable prognosis in patients with BLCA, CHOL, and KIRC (**Figures 2J–L**). These results suggest that SLC12A7 plays distinct roles in different cancer types. In tumors such as LIHC, LGG, and GBM, high SLC12A7 expression may contribute to tumor progression, thereby acting as a risk factor. In contrast, in cancers like BLCA and KIRC, its elevated expression may reflect a compensatory mechanism or involvement in anti-tumor immune responses, serving as a protective factor. These findings highlight the functional complexity of SLC12A7 in tumor biology and emphasize the importance of tumor-specific regulatory networks.

### 3.3 The correlation between SLC12A7, immune cell infiltration, and immune checkpoints

**Figure 3:**
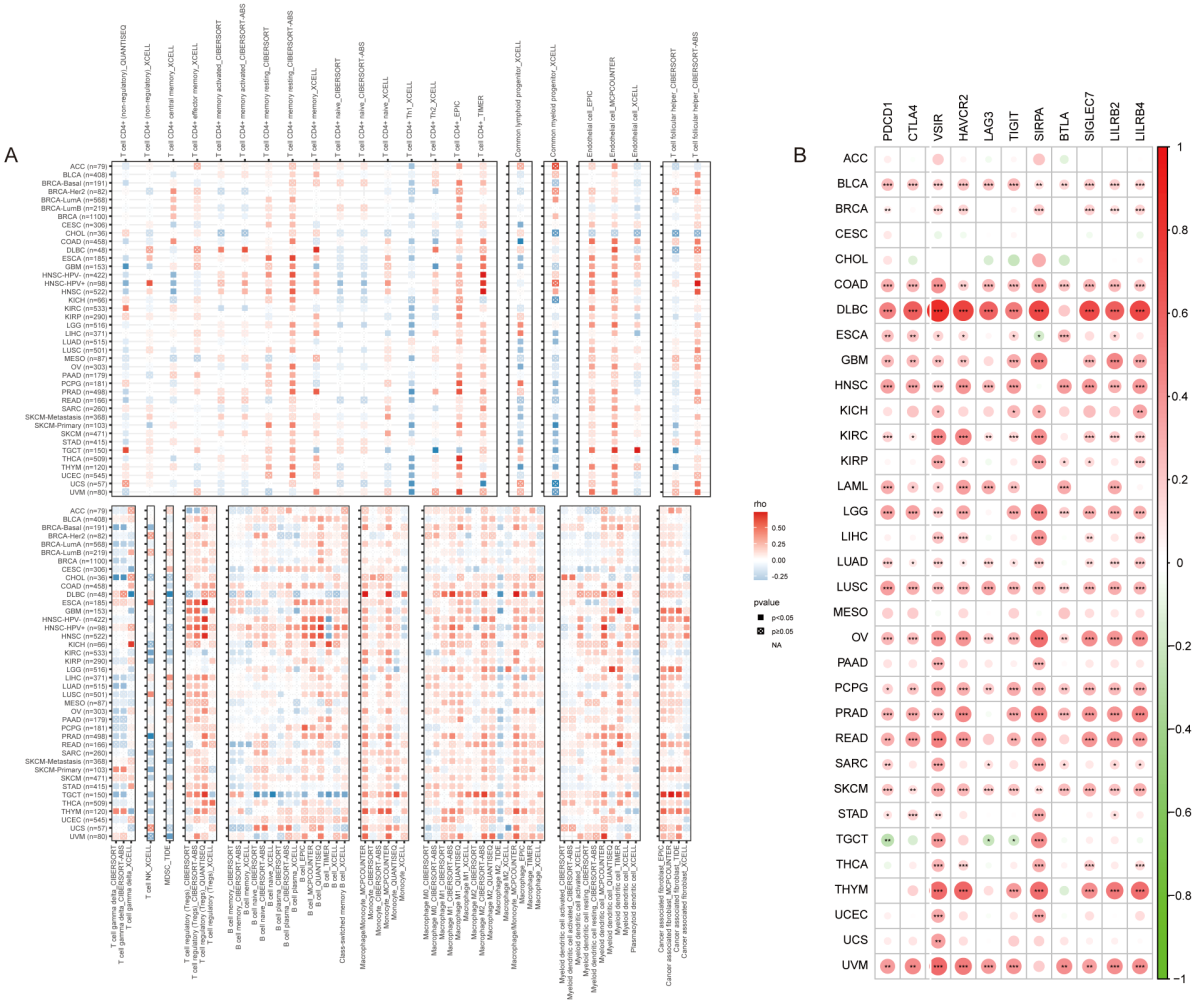
Correlation of SLC12A7 with Immune Cell Infiltration and Immune Checkpoints. **(A)** The correlation heatmap shows the relationship between SLC12A7 expression and the infiltration of various immune cells. The color scale from blue to red represents the correlation coefficient. **(B)** The heatmap shows the correlation between SLC12A7 expression and 11 immune checkpoint genes. The color scale from blue to red represents the correlation coefficient. *p < 0.05; **p < 0.01; ***p < 0.001.

To evaluate the role of SLC12A7 in pan-cancer immunity, we systematically analyzed the relationships between SLC12A7 expression and immune cell infiltration using methods such as XCELL, TIMER, and CIBERSORT. In multiple cancers, increased SLC12A7 expression was accompanied by a significant decrease in T cell gamma delta and NK T cell infiltration (**Figure 3A**). Meanwhile, SLC12A7 showed significant positive correlations with stromal and myeloid cell infiltration across various cancers, especially cancer-associated fibroblasts, dendritic cells, and macrophages (**Figure 3A**). These results suggest a potential role of SLC12A7 in immune regulation and matrix remodeling.

To further explore the association between SLC12A7 and tumor immunity, we analyzed the correlations between SLC12A7 expression and multiple immune checkpoints. The results showed that SLC12A7 was significantly associated with immune checkpoints across different cancer types (**Figure 3B**). Specifically, SLC12A7 was significantly positively correlated with CTLA4, PDCD1, TIGIT, VSIR, SIRPA, and HAVCR2 in many cancers, indicating a potential role of SLC12A7 in tumor immune evasion (**Supplementary Figures S1A–F**). In addition, we assessed the correlations between SLC12A7 expression and tumor mutational burden (TMB) and microsatellite instability (MSI), both of which have been linked to sensitivity to immune checkpoint inhibitors (ICIs) in multiple studies. SLC12A7 expression was positively correlated with TMB in CHOL, ESCA, KIRP, and UCEC, but negatively correlated with TMB in OV (**Supplementary Figure S1G**). At the same time, SLC12A7 expression was positively correlated with MSI in CESC and LIHC, but negatively correlated with MSI in UCS and COAD (**Supplementary Figure S1H**). These findings suggest that SLC12A7 may participate in shaping the tumor immune microenvironment, possibly by modulating immune cell infiltration and immune checkpoint expression. Its positive correlations with key inhibitory immune checkpoints and ICI-related biomarkers (such as TMB and MSI) further imply a role for SLC12A7 in immune evasion and responses to immune checkpoint blockade. Nevertheless, although the above analyses to some extent reveal associations between SLC12A7 and tumor immune features, they do not establish SLC12A7 as a predictive biomarker for ICI efficacy and still require further validation in ICI clinical cohorts with clearly defined treatment outcomes.

### 3.4 The role of cancer-related pathways involving SLC12A7

**Figure 4:**
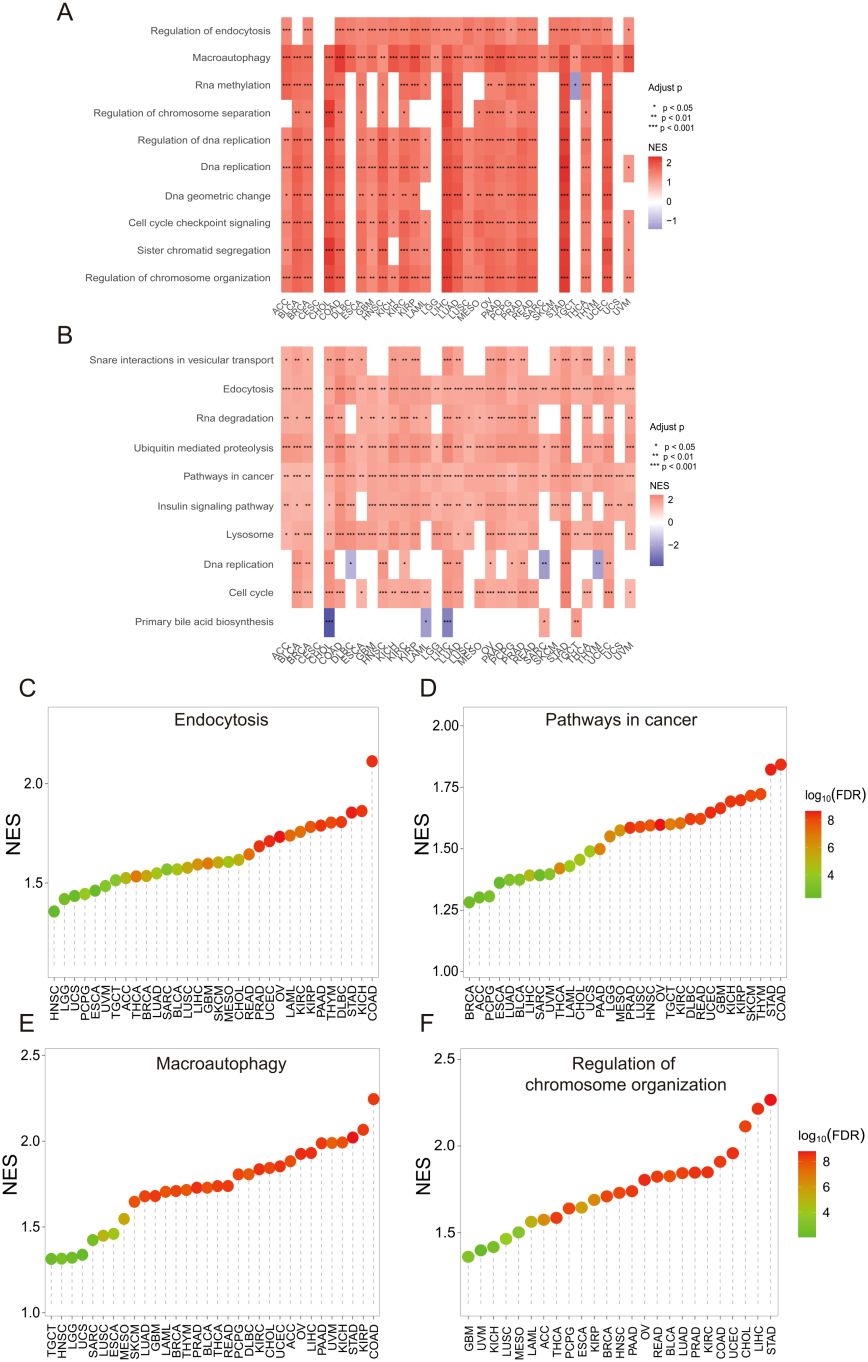
SLC12A7 is associated with cancer-related pathways and biological processes. **(A)** A heatmap based on GSEA showing biological processes related to SLC12A7, with the color scale from blue to red representing NES values. **(B)** The heatmap shows Kyoto Encyclopedia of Genes and Genomes pathways associated with SLC12A7 based on GSEA. The color scale from blue to red represents the NES values. **(C)** Endocytosis, **(D)** Pathways in cancer, **(E)** Macroautophagy, and **(F)** Regulation of chromosome organization, ranked by NES values across 32 cancer types. GSEA, Gene Set Enrichment Analysis; NES, normalized enrichment score.

To predict the potential function and mechanism of SLC12A7 in pan-cancer, Gene Set Enrichment Analysis (GSEA) was performed to enrich SLC12A7-related Kyoto Encyclopedia of Genes and Genomes (KEGG) pathways and biological processes. The GSEA results for biological processes suggested that SLC12A7 might be potentially involved in various biological processes in certain cancer types, including regulation of endocytosis, macroautophagy, regulation of DNA replication, cell cycle checkpoint signaling, and regulation of chromosome organization (**Figure 4A**). These biological processes play crucial roles in the initiation and progression of cancer. Additionally, the GSEA results for KEGG pathways revealed significant enrichment of several cancer-related pathways, such as the cell cycle, DNA replication, pathways in cancer, and endocytosis (**Figure 4B**). Meanwhile, we analyzed the enrichment scores of several cancer-related biological processes and KEGG pathways across 32 cancer types. The results showed that the Normalized Enrichment Score (NES) for endocytosis, pathways in cancer, and macroautophagy was highest in COAD (**Figures 4C–E**). The NES for regulation of chromosome organization was significantly higher in LIHC and STAD compared to other cancer types (**Figure 4F**). Together, these findings suggest that dysregulated SLC12A7 expression may promote tumor progression by enhancing proliferative signaling, disrupting genomic maintenance, and facilitating cellular plasticity across diverse cancer types.

### 3.5 Heterogeneity and potential function of SLC12A7 in the hepatocellular carcinoma microenvironment

To investigate the expression heterogeneity of SLC12A7 within the tumor microenvironment (TME), we leveraged the TISCH database to examine its expression patterns across 12 cancer types (**Supplementary Figures S2A–L**). The results showed a consistent distribution trend of SLC12A7 across multiple tumor types, with predominant enrichment in fibroblasts, epithelial cells, and malignant cells (**Supplementary Figures S2A–L**).

**Figure 5:**
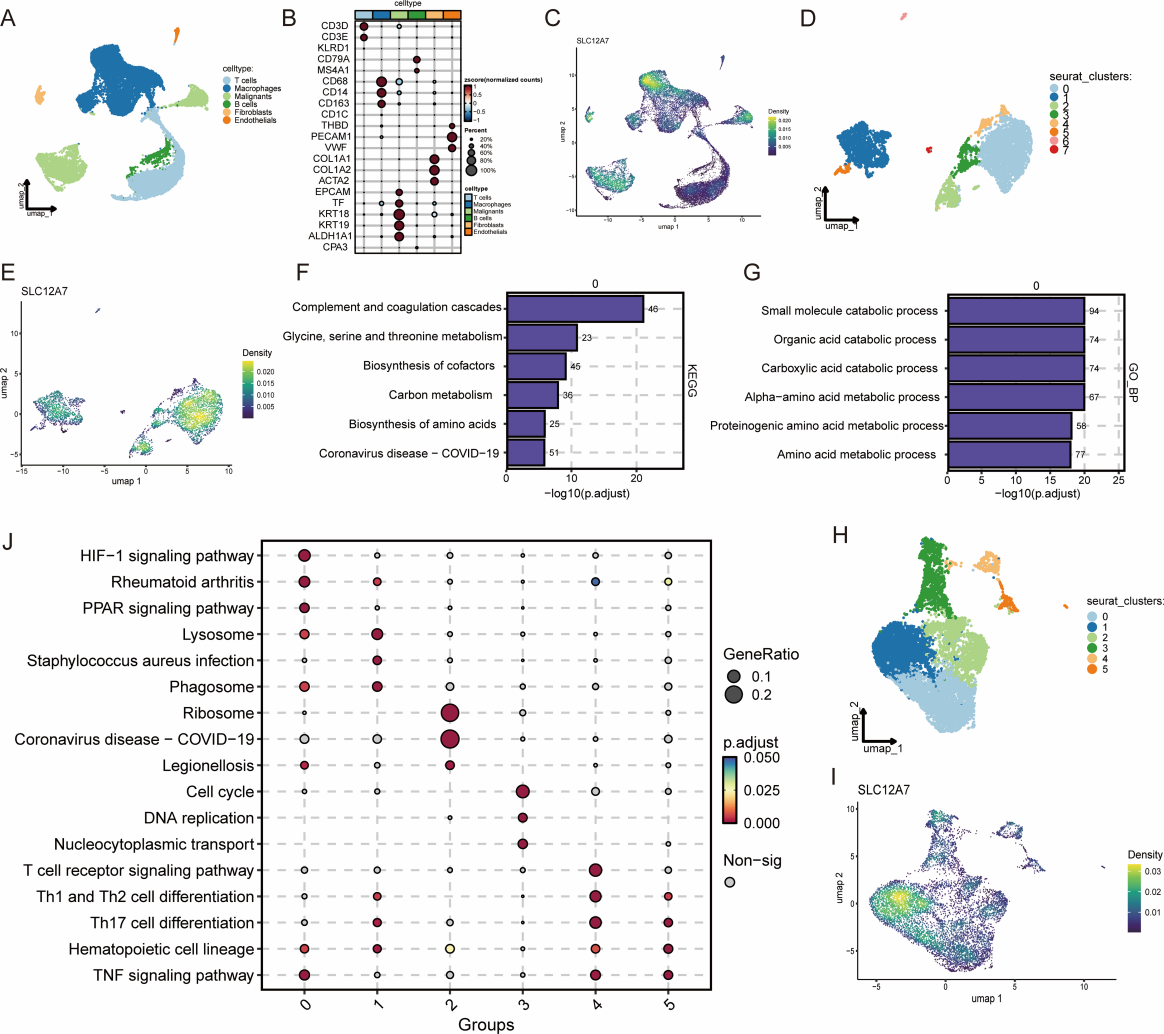
Expression heterogeneity of SLC12A7 in the HCC tumor microenvironment and its associated functional features. **(A)** UMAP plot showing 6 major cell types. **(B)** Canonical marker genes used to validate cell-type annotation. **(C)** Density distribution of SLC12A7 expression across TME cell populations. **(D)** Malignant cells were further subdivided into 8 clusters (UMAP). **(E)** Density distribution of SLC12A7 expression across malignant cell subclusters. **(F, G)** KEGG and GO enrichment analyses of malignant cell cluster 0. **(H)** Macrophages were further subdivided into 7 clusters (UMAP). **(I)** Density distribution of SLC12A7 expression across macrophage subclusters. **(J)** KEGG enrichment analysis across macrophage subclusters.

To further elucidate the functional role of SLC12A7 in the HCC TME, we retrieved single-cell RNA-sequencing data of HCC from the GEO database (16). After stringent quality control, 6 major cell types were retained, including T cells, macrophages, malignant cells, B cells, fibroblasts, and endothelial cells (**Figure 5A**). The annotation accuracy was supported by canonical specific marker genes (**Figure 5B**). We first assessed SLC12A7 expression within the TME. Notably, SLC12A7 was highly enriched in malignant cells and macrophages (**Figure 5C**). However, we also observed pronounced intratumoral heterogeneity of SLC12A7 expression, as not all malignant cells exhibited uniformly high expression. To characterize the functional features of the SLC12A7-high tumor subpopulation, malignant cells were further stratified into 8 subclusters (**Figure 5D**), and SLC12A7 was found to be significantly upregulated in the cluster0 (**Figure 5E**). Consistently, GO and KEGG based enrichment analyses indicated that the c0 subcluster displayed prominent metabolism-associated features (**Figures 5F, G**), suggesting a potential role of SLC12A7 in tumor metabolic reprogramming. In addition, macrophages were further divided into 7 clusters (**Figure 5H**), and SLC12A7 was significantly enriched in the cluster1 (**Figure 5I**). Enrichment analyses across distinct macrophage subclusters showed that, compared with other macrophage subclusters, the cluster1 subcluster exhibited a stronger phagocytic state (**Figure 5J**). These findings suggest a potential role of SLC12A7 in macrophage-mediated antitumor immunity.

### 3.6 Genetic alterations of SLC12A7

**Figure 6:**
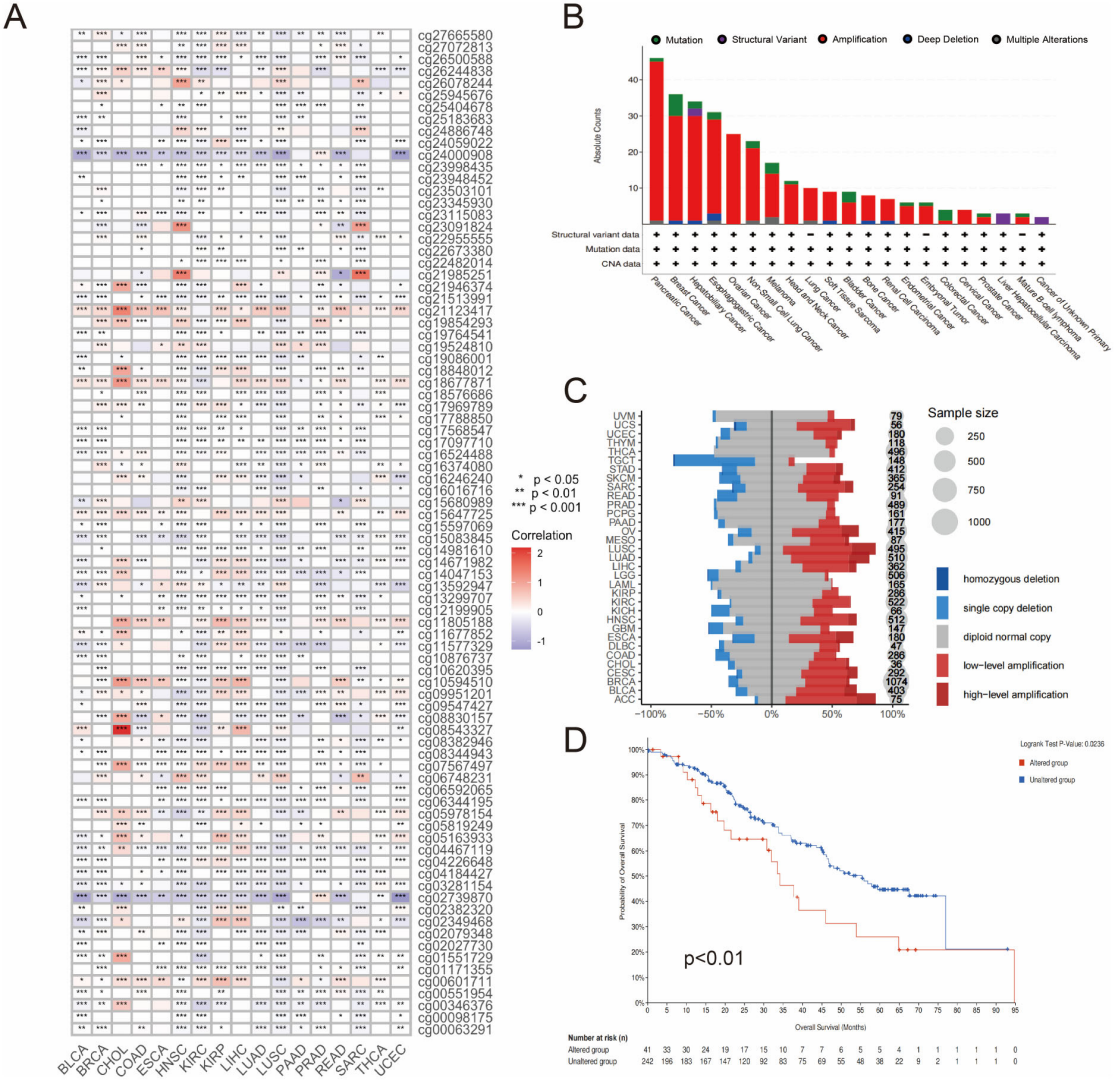
Genetic alterations of SLC12A7. **(A)** Correlation analysis between pan-cancer DNA methylation and SLC12A7 expression based on the TCGA database. **(B)** Mutation counts and mutation types of SLC12A7 in 21 cancer types according to cBioPortal database. **(C)** DNA copy number variation analysis of SLC12A7 across pan-cancer types based on the TCGA database. **(D)** Kaplan-Meier overall survival (OS) curve for SLC12A7 gene alterations based on the cBioPortal database.

To elucidate the mechanisms underlying the increased expression of SLC12A7, the methylation, mutations, and copy number variations (CNVs) of SLC12A7 were analyzed. The results showed that at the epigenetic level, methylation of specific sites in the SLC12A7 promoter region was associated with mRNA expression levels across various cancer types. Notably, methylation of cg24000908 and cg02739870 was significantly negatively correlated with mRNA expression in BLCA, CHOL, COAD, ESCA, HNSC, KIRC, KIRP, LIHC, LUAD, LUSC, READ, and UCEC (**Figure 6A**). Furthermore, amplification and mutations were identified as the major genetic alterations of SLC12A7. Pancreatic cancer, breast cancer, hepatobiliary cancer, esophagogastric cancer, and ovarian cancer had the highest number of genetic alterations (**Figure 6B**). In most cancer types, low-level amplification was the most common copy number variation (CNV) pattern for SLC12A7, whereas in TGCT, LGG, and GBM, the primary alteration was single copy deletion (**Figure 6C**). Finally, we examined the impact of SLC12A7 genomic alterations on patient prognosis, and the results revealed that patients with SLC12A7 genetic alterations had significantly worse prognoses compared to those without alterations (**Figure 6D**). These findings suggest that the aberrant expression of SLC12A7 may be driven by a combination of epigenetic and genomic alterations. Promoter hypomethylation at specific CpG sites may enhance transcriptional activity by loosening chromatin structure or disrupting the binding of repressive transcription factors. In parallel, gene amplification could increase the copy number and expression levels of *SLC12A7*, thereby potentially enhancing its role as a membrane transporter involved in ion homeostasis and cell volume regulation—processes that are known to facilitate cell proliferation, migration, and interactions with the tumor microenvironment. Collectively, these alterations may contribute to tumor progression and poor clinical outcomes in cancer patients.

### 3.7 SLC12A7 enhances proliferation and migration of hepatocellular carcinoma cells

**Figure 7:**
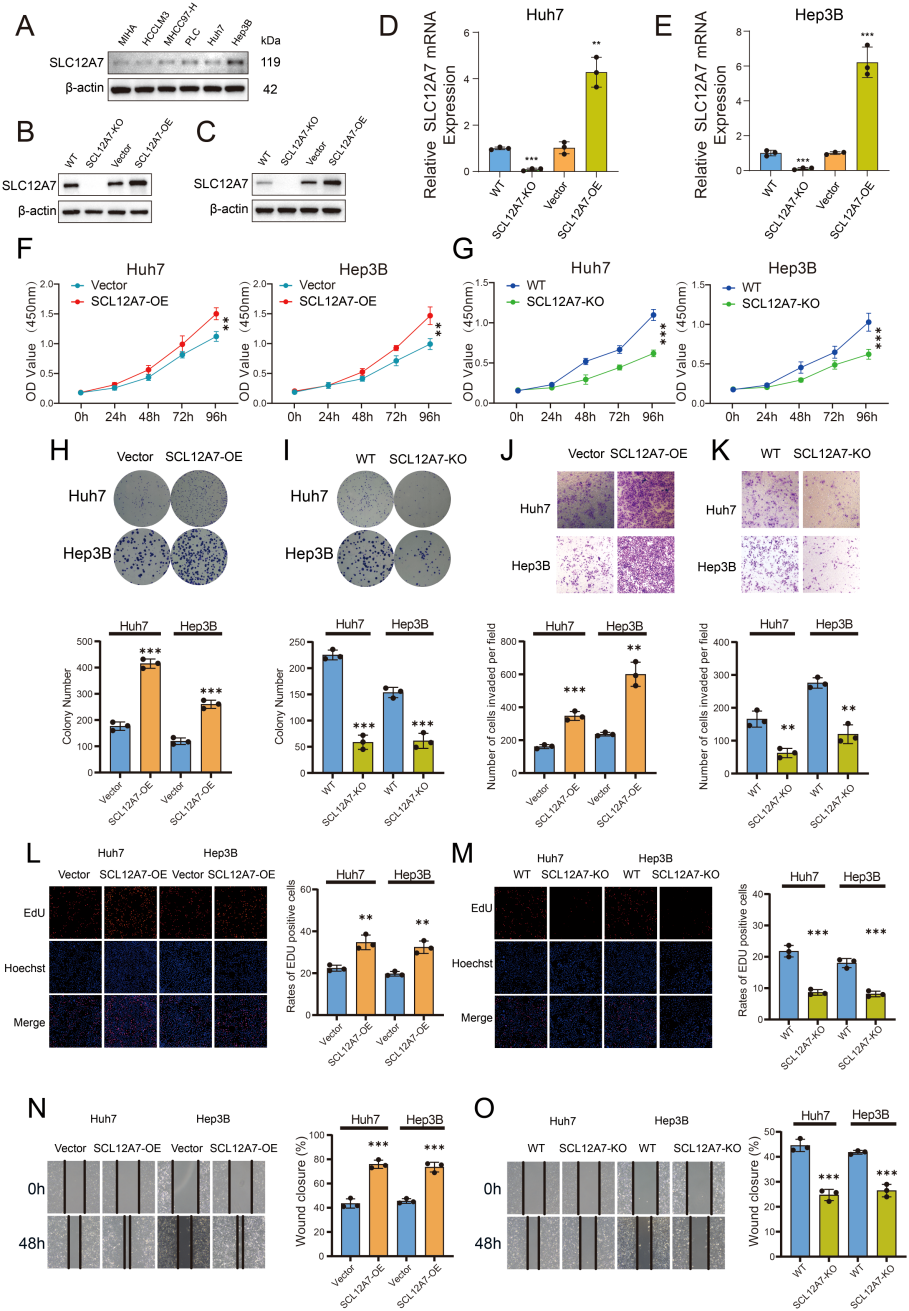
Functional characterization of SLC12A7 modulation in HCC cells. **(A)** WB analysis showing a significant increase in SLC12A7 expression in HCC cells. **(B-E)** Validation of SLC12A7 expression at both the protein and RNA levels. **(F, G)** CCK-8 viability assays demonstrating the impact of SLC12A7 overexpression **(F)** or knockout **(G)** on Huh7 and Hep3B cell proliferation over 96 hours. **(H, I)** Colony formation analysis revealed significant differences in clonogenic potential between SLC12A7-overexpressing **(H)** and knockout **(I)** groups compared to controls. **(J, K)** Transwell assays demonstrated that SLC12A7 overexpression significantly enhanced, whereas SLC12A7 knockout markedly suppressed, the migratory capacity of HCC cells. **(L, M)** EdU incorporation assays quantified proliferative activity, with representative fluorescence micrographs showing EdU^+^ cells (red) counterstained with DAPI (blue). **(N, O)** Scratch wound healing assays demonstrated accelerated or impaired migratory dynamics in SLC12A7-overexpressing **(N)** versus knockout **(O)** cohorts at 24-hour intervals. Data are expressed as mean ± SEM from three independent experiments. Statistical significance was determined by two-tailed Student’s t-test: ***p < 0.001, **p < 0.01, *p < 0.05; n.s., not significant.

To validate the findings obtained from public database analyses, we conducted cellular experiments focusing on SLC12A7. Western blot (WB) analysis was performed to evaluate the expression levels of SLC12A7 in normal human hepatocytes and various hepatocellular carcinoma (HCC) cell lines (**Figure 7A**). The results revealed that SLC12A7 expression was markedly upregulated in HCC cells. Subsequently, we established HCC cell lines with stable overexpression or knockout of SLC12A7 and verified the expression levels at both the protein and RNA levels (**Figures 7B–E**).

To investigate the functional role of SLC12A7 in HCC cell biology, a series of *in vitro* assays were systematically conducted to assess its effects on cell proliferation and migration in Huh7 and Hep3B cell lines. CCK-8 assays demonstrated that stable overexpression of SLC12A7 significantly enhanced HCC cell proliferation (**Figure 7F**), whereas SLC12A7 knockout notably suppressed cell viability (**Figure 7G**). Colony formation assays further confirmed that SLC12A7 overexpression markedly promoted the colony-forming ability of Huh7 and Hep3B cells (**Figure 7H**), whereas SLC12A7 knockout significantly impaired this capacity (**Figure 7I**). To further evaluate the impact of SLC12A7 on HCC cell migration, Transwell and wound healing assays were performed. Transwell results showed that SLC12A7 overexpression significantly enhanced the migration capacity of Huh7 and Hep3B cells (**Figure 7J**), while its knockout substantially inhibited their potential of migration (**Figure 7K**). EdU incorporation assays indicated that SLC12A7 overexpression significantly increased the proliferation index of HCC cells (**Figure 7L**), whereas its knockout led to reduced proliferative activity (**Figure 7M**). Consistently, wound healing assays revealed that SLC12A7 overexpression facilitated cell migration (**Figure 7N**), while SLC12A7 knockout markedly suppressed the migratory ability of HCC cells (**Figure 7O**).

Statistical analyses confirmed that SLC12A7 overexpression significantly promoted tumor-related cellular behaviors, whereas its depletion resulted in a marked reduction in cell viability and aggressiveness (P < 0.05). These findings suggest that SLC12A7 plays a pivotal oncogenic role in hepatocellular carcinoma by facilitating key tumorigenic processes such as cell proliferation and migration. As a membrane transporter involved in ion transport and cell volume regulation, elevated SLC12A7 expression may enhance intracellular ionic homeostasis and osmotic balance, thereby creating a favorable microenvironment for cancer cell growth and motility. Collectively, these results support the hypothesis that SLC12A7 contributes to HCC progression by modulating critical cellular behaviors essential for tumor invasion.

## 4 Discussion

Cancer remains one of the leading causes of death worldwide, and HCC ranks among the major contributors to cancer-related mortality globally (17). Although diagnostic and therapeutic strategies for HCC have been continuously updated in recent years, the overall clinical benefit remains limited (18). Therefore, the continued identification of novel prognostic biomarkers and actionable targets is still warranted. SLC12A7 (KCC4) belongs to the K^+^/Cl^−^ cotransporter family, whose canonical functions are related to ion homeostasis and cell-volume regulation (19–21). Previous studies in cervical and ovarian cancer models have shown that IGF-1 can activate K^+^/Cl^−^ cotransport, and that KCC activity is critical for cancer cell invasion and proliferation (22). Pharmacological inhibition or genetic interference of KCC activity can markedly attenuate these malignant phenotypes. In addition, membrane trafficking and membrane localization of KCC4 are considered key prerequisites for its pro-invasive effects, and related work suggests that promoting KCC4 recruitment to the plasma membrane can enhance tumor cell invasiveness (5, 6). In adrenocortical carcinoma, SLC12A7 exhibits amplification and overexpression and can promote a more invasive tumor behavior by altering cellular adhesion properties (23). However, existing evidence has largely focused on individual cancer types or specific phenotypes, and systematic analyses of SLC12A7 expression and prognostic features at the pan-cancer level, as well as its association with the immune microenvironment, remain limited. Meanwhile, in the HCC tumor microenvironment, the expression heterogeneity of SLC12A7 and its potential functions require further clarification. Based on these considerations, the present study integrates multi-omics analyses with *in vitro* functional experiments, thereby advancing the understanding of the oncological significance of SLC12A7.

Through pan-cancer analyses of TCGA data, we found that SLC12A7 is highly expressed in multiple tumor tissues. Moreover, increases at the level of certain protein-coding transcripts and protein products were concordant with the direction of mRNA changes, suggesting that its upregulation may be influenced by transcriptional regulation or genetic alterations. Univariate Cox analysis indicated that high SLC12A7 expression is associated with unfavorable prognosis in several cancer types, and Kaplan–Meier analysis further supported this finding. Overall, these results support that SLC12A7 can significantly affect prognosis in pan-cancer patients, suggesting its potential as a candidate prognostic indicator in specific cancer types. Given the marked upregulation of SLC12A7 in HCC and its impact on clinical outcomes, we further assessed its expression in cell lines. The results showed that SLC12A7 protein levels were markedly higher in multiple HCC cell lines than in the normal liver cell line MIHA, suggesting that SLC12A7 may participate in HCC initiation and progression and may have potential functional relevance.

Meanwhile, we characterized the expression heterogeneity of SLC12A7 within the HCC tumor microenvironment, with predominant expression in malignant cells and macrophages. The malignant cell subpopulation with high SLC12A7 expression exhibited stronger metabolism-associated features, and SLC12A7 was also significantly enriched in a macrophage subpopulation with more prominent phagocytic function. The composition and functional states of immune cells are important components of the TME, and the tumor immune microenvironment (TIME) can profoundly influence tumor progression and responses to immunotherapy (24, 25). In this study, across multiple cancer types, high SLC12A7 expression was negatively correlated with the infiltration of immune cell populations involved in cytotoxicity, such as NK T cells, whereas it was positively correlated with the infiltration of multiple stromal cell populations, including cancer-associated fibroblasts, in many cancers. NK T cells play a critical role in antitumor immunity and are typically associated with a more favorable prognosis (26). In contrast, CAFs can promote immunosuppression through multiple mechanisms and drive tumor initiation, progression, and metastasis (27–29). Therefore, our findings suggest that high SLC12A7 expression may be more likely to correspond to a TIME state with immunosuppressive or immune-excluded features. Furthermore, SLC12A7 expression showed significant positive correlations with multiple immune checkpoint genes across various cancer types. Immune checkpoint pathways represent a central mechanism of tumor immune evasion, and their blockade has become an important antitumor strategy (30). However, a substantial proportion of patients still exhibit resistance or limited benefit from immunotherapy in clinical practice (31). Tumor mutational burden (TMB) and microsatellite instability (MSI), as pan-cancer biomarkers for predicting responses to immune checkpoint inhibitors (ICI), have demonstrated predictive value in multiple cancers (32, 33). We observed significant correlations between SLC12A7 and TMB/MSI across multiple cancer types, suggesting that high SLC12A7 expression may be associated with genomic instability, potential changes in neoantigen load, and immune landscape remodeling, thereby potentially influencing responses to immunotherapy. It should be emphasized, however, that the present study does not include ICI-treated real-world cohorts or treatment response data. Therefore, the current findings are correlation-based and further validation in independent ICI-treated clinical cohorts with clearly defined response endpoints is warranted.

At the genetic and epigenetic levels, we performed pan-cancer analyses of SLC12A7 methylation, mutations, and copy number variations. First, we found that the methylation levels of certain sites in the promoter region of SLC12A7 were significantly negatively correlated with mRNA expression in 12 cancer types, suggesting that epigenetic regulation may represent an important mechanism underlying aberrant SLC12A7 upregulation in tumors. Second, copy number amplification and mutation were the major forms of SLC12A7 alterations. Further prognostic analyses also supported associations between SLC12A7 genomic alterations and clinical outcomes. To explore the potential functional mechanisms involving SLC12A7, we performed functional enrichment analyses. GSEA results suggested that SLC12A7 may be associated with multiple tumor-related biological processes, including pathways such as macroautophagy and endocytosis. Macroautophagy is considered to support tumor growth by promoting metabolic adaptation, stress tolerance, and immune evasion (34, 35). Meanwhile, SLC12A7-related gene sets were also enriched in proliferation-associated processes such as the cell cycle and DNA replication, which may be consistent with tumor progression. It should be noted that enrichment analyses reflect statistical co-variation, and causal mechanisms still require further clarification through pathway perturbation, assays of ion-transport activity, and validation of key downstream molecules.

To validate the specific function of SLC12A7 in HCC, we established and verified HCC cell models with SLC12A7 knockout and overexpression, and confirmed effective construction at both the protein and RNA levels. CCK-8, colony formation, and EdU assays showed that SLC12A7 overexpression markedly promoted HCC cell proliferation *in vitro*, whereas knockout reduced proliferative capacity. Transwell and wound-healing assays further demonstrated that SLC12A7 overexpression significantly enhanced HCC cell invasion and migration, while knockout exerted the opposite effect. These functional experiments support a tumor-promoting role of SLC12A7 in HCC at the cellular phenotype level, consistent with the progression-associated signals observed in our pan-cancer and single-cell analyses.

It should be emphasized that, although previous studies have indicated that SLC12A7 is associated with invasive phenotypes in multiple cancer types, the contribution of the present work lies in investigating the expression and functional characteristics of SLC12A7 in a pan-cancer context and characterizing its associations with TIME alterations. Nevertheless, this study has certain limitations. First, the immune infiltration analyses were primarily based on computational inference, lacking validation using real-world ICI-treated cohorts with efficacy endpoints. In addition, the mechanisms by which SLC12A7 drives metabolic reprogramming or immune regulation through ion transport, membrane localization, and downstream signaling pathways still require more in-depth mechanistic experiments and *in vivo* model studies. Future studies in larger clinical cohorts and with multidimensional functional validation are needed to further clarify the clinical utility and druggability potential of SLC12A7.

## 5 Conclusion

This study demonstrates the expression, prognostic value, immune relevance, biological function, genetic alterations, and single-cell expression distribution of SLC12A7 across multiple cancers, with experimental validation in HCC. Our findings reveal that SLC12A7 is aberrantly expressed in various cancers and significantly impacts patient prognosis. SLC12A7 may contribute to tumor immune escape and influence the immune microenvironment. Moreover, its overexpression promotes the proliferation and metastasis of HCC cells. Therefore, SLC12A7 should not be regarded solely as a regulator of ion homeostasis but rather as a potential biomarker associated with multiple cancers, playing a crucial role in tumorigenesis and progression.

## Data Availability

The original contributions presented in the study are included in the article/**Supplementary Material**. Further inquiries can be directed to the corresponding authors.
