# Supplementary material for: SLC12A7 serves as a prognostic and immunotherapeutic biomarker identified by multi-omics analysis
Source: Front Immunol. 2026 Jan 26;16:1712579. doi: 10.3389/fimmu.2025.1712579 (PMC12883764; doi:10.3389/fimmu.2025.1712579)
Supplement: Supplementary file 1 [file DataSheet1.pdf]

Supplementary Material

Fig. S1

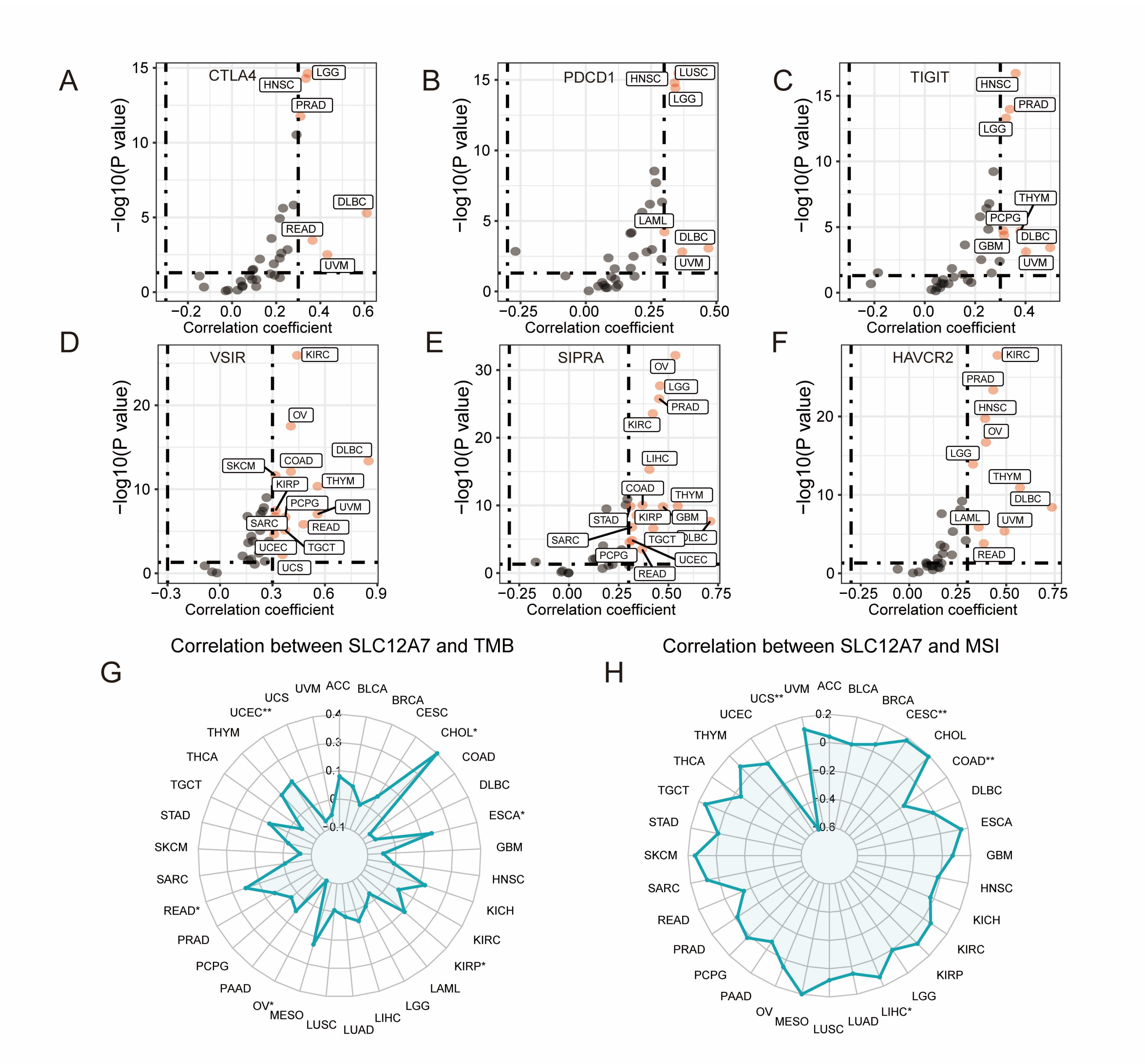

Pan-Cancer Correlation of SLC12A7 with Immunity, TMB, and MSI. A-F Scatter plots display the Spearman correlation analysis results between SLC12A7 expression and the expression of (A) CTLA4, (B) PDCD1, (C) TIGIT, (D) VSIR, (E) SIRPA, and (F) HAVCR2 across pan-cancer. G-H The correlation between SLC12A7 expression and (G) microsatellite instability (MSI) and (H) tumor mutational burden (TMB) across pan-cancer.

Fig. S2

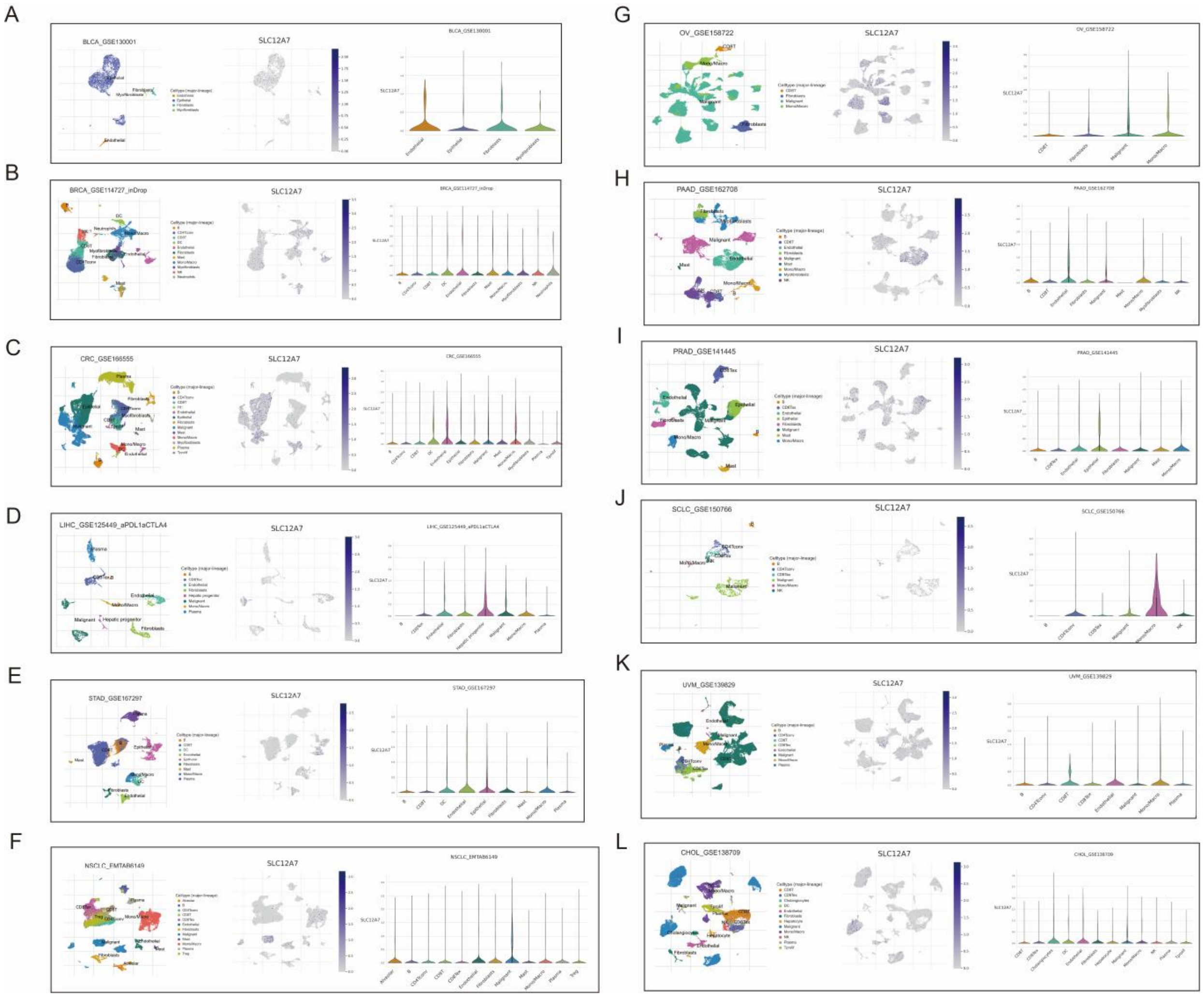

scRNA-seq Pan-Cancer Analysis of SLC12A7. A-L Expression distribution and heterogeneity of SLC12A7 across Single-Cell landscapes of different cancers, including BLCA, BRCA, CRC, LIHC, STAD, NSCLC, OV, PAAD, PRAD, SCLC, UVM and CHOL: UMAP plots reveal cellular distribution and heterogeneous SLC12A7 expression in various tumors
